# Supplementary figures and images for: Microbial communities inhabiting the surface and gleba of white (Tuber magnatum) and black (Tuber macrosporum) truffles from Russia
Source: PeerJ. 2025 Sep 19;13:e20037. doi: 10.7717/peerj.20037 (PMC12452945; doi:10.7717/peerj.20037)

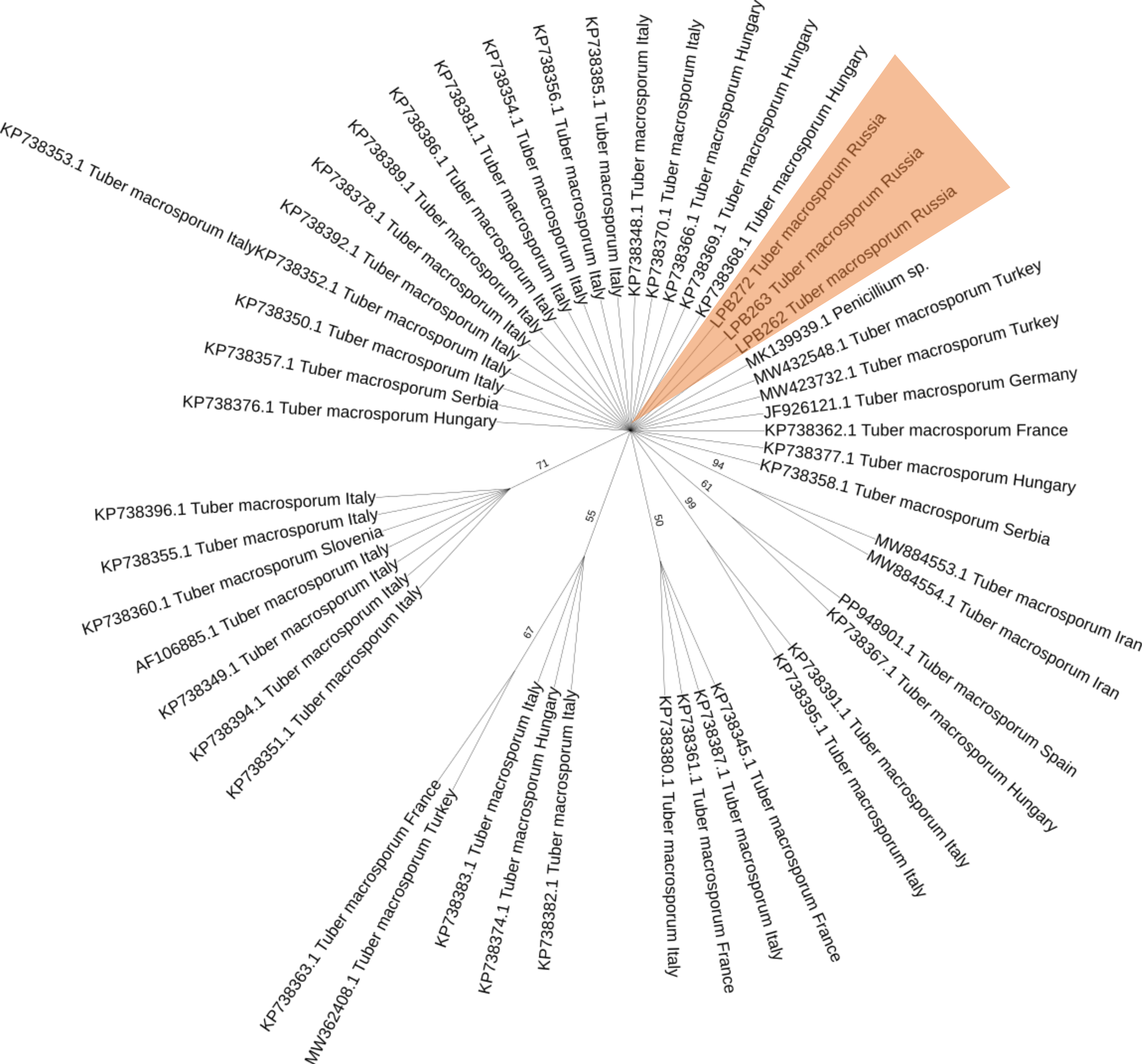

Supplement: Supplemental Information 1 [file peerj-13-20037-s001.png]

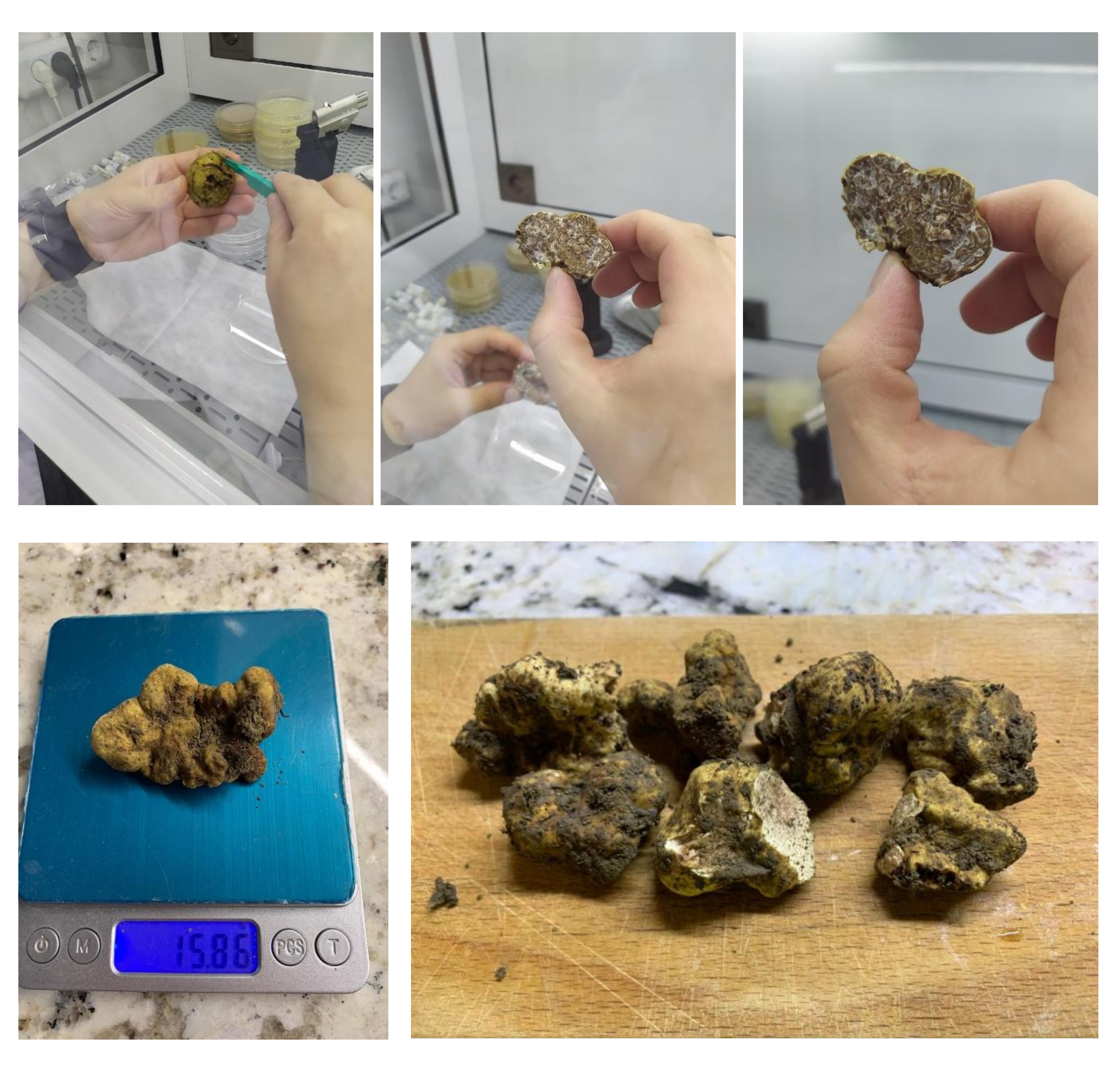

Supplement: Supplemental Information 2 [file peerj-13-20037-s002.jpg]

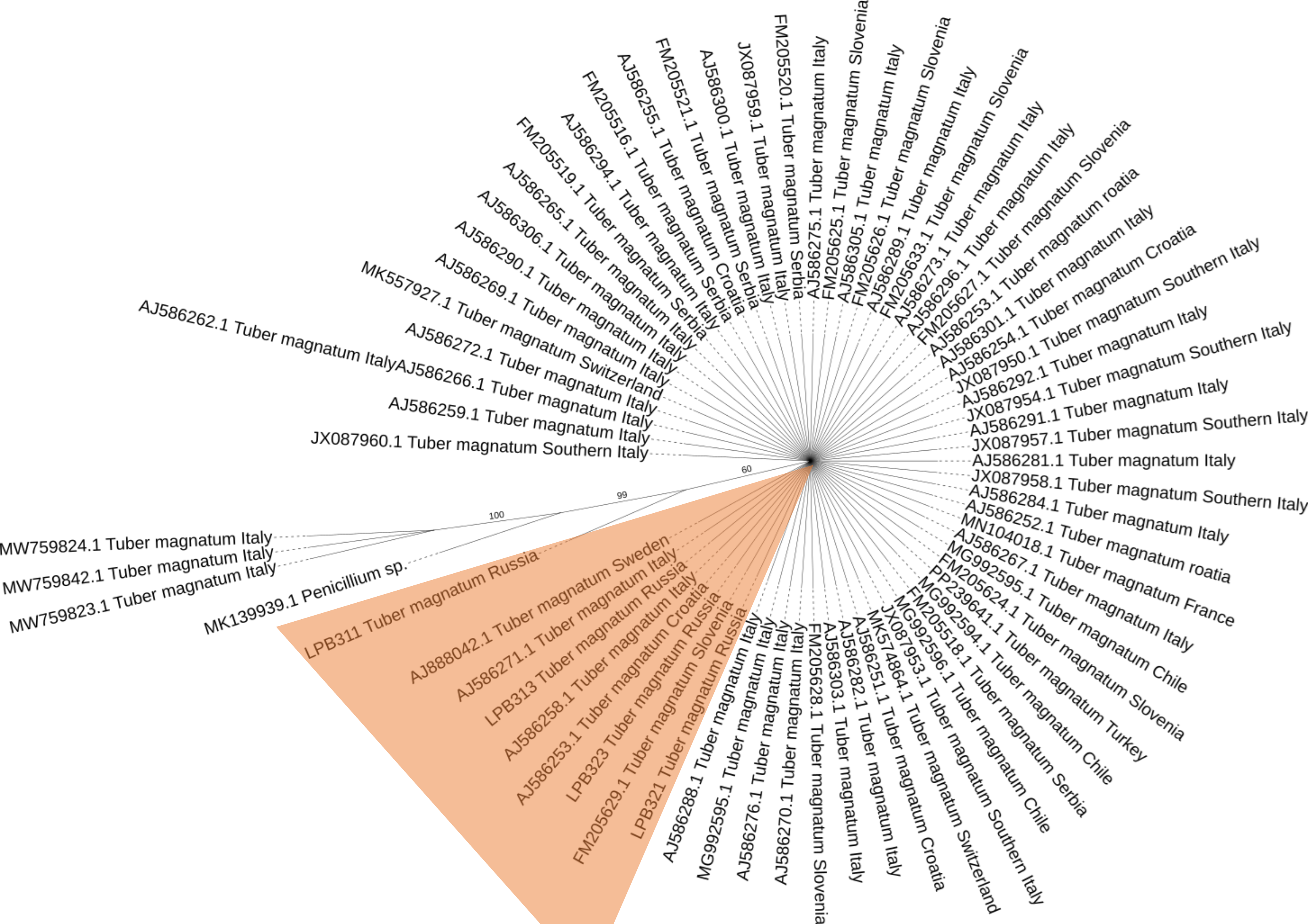

Supplement: Supplemental Information 3 [file peerj-13-20037-s003.png]

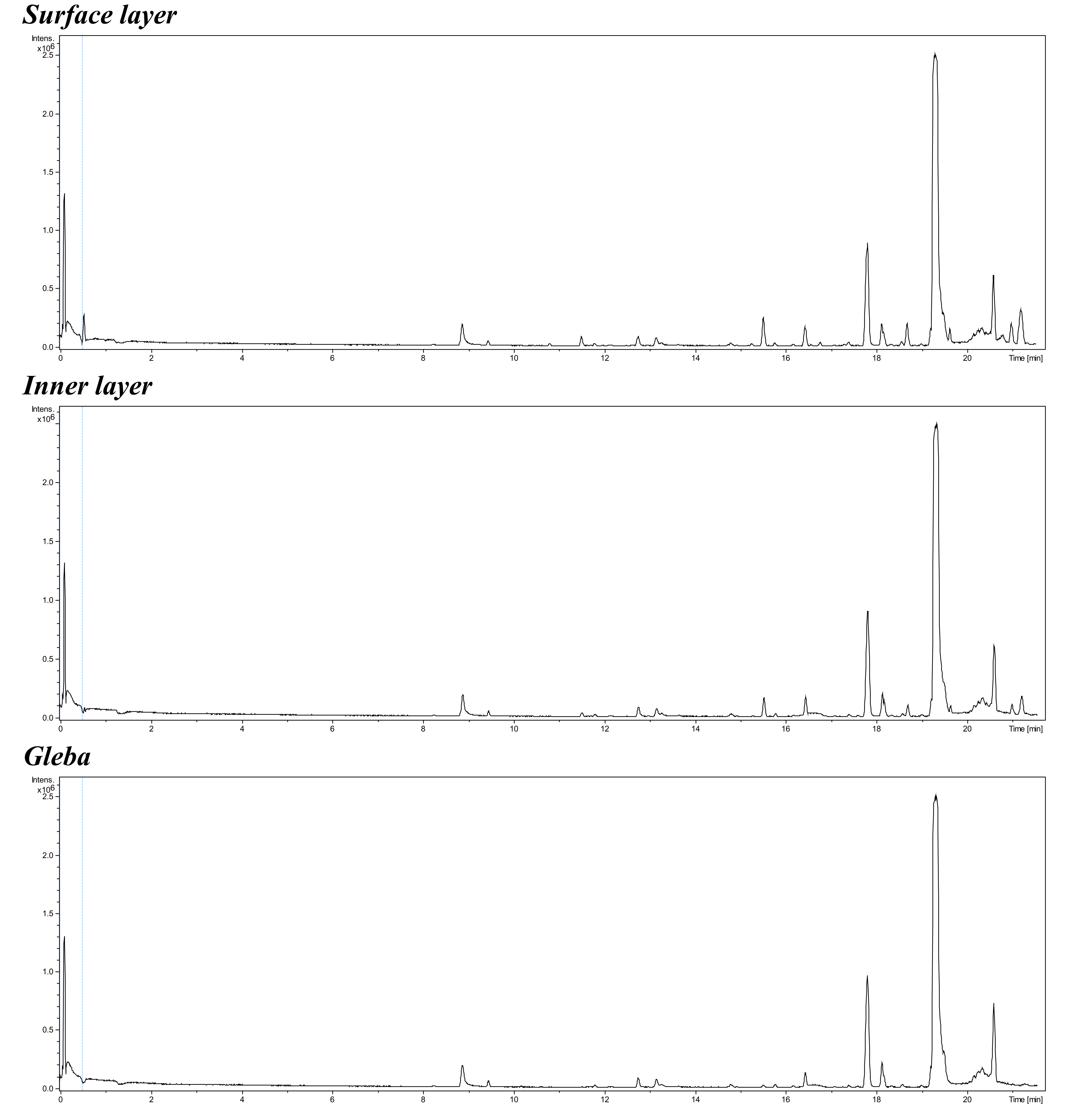

Supplement: Supplemental Information 4 — For the extraction, 0.5 g of each truffle tissue sample was ground into a powder using a mortar and pestle with the addition of acetonitrile in a 1:10 ratio (w/v; weight of tissue to volume of solvent). The resulting homogenate was centrifuged for 10 min at 3,000× g rpm (Armed LC04B, St. Petersburg, Russia). Then, 800 µL of the supernatant was transferred into microtubes, and protein precipitation was performed by adding 80 µL of a 10% trichloroacetic acid solution. The microtubes were centrifuged again for 10 min at 16,000× g rpm (Biosan Microspin-12, Riga, Latvia). The prepared samples were stored at +4 °C. Prior to analysis, the samples were filtered through a 13 mm syringe filter with 45 µm pores and a polyvinylidene fluoride (PVDF) membrane, after which 100 µL of the filtrate was transferred into chromatographic vials. The extracts were then diluted with 900 µL of acetonitrile. Chromatographic determination was performed using ultra-high-performance liquid chromatography coupled with a triple quadrupole mass spectrometer (6470, Agilent Technologies, Germany) and an analytical Zorbax SB-Aq column (4.6 × 50 mm, 5 µm). The chromatographic conditions were set using deionized water/formic acid (99.9:0.1, v/v) (solvent A) and methanol/formic acid (99.9:0.1, v/v) (solvent B). The chromatographic separation of target compounds was carried out according to the following gradient (time (minutes), %B): (0, 30%); (3, 10%); (4, 30%). The injection volume and flow rate were 3 µL and 0.8 mL/min, respectively. Data acquisition and processing were performed using MassHunter software version B.08.00 (Agilent Technologies, Germany). [file peerj-13-20037-s004.png]

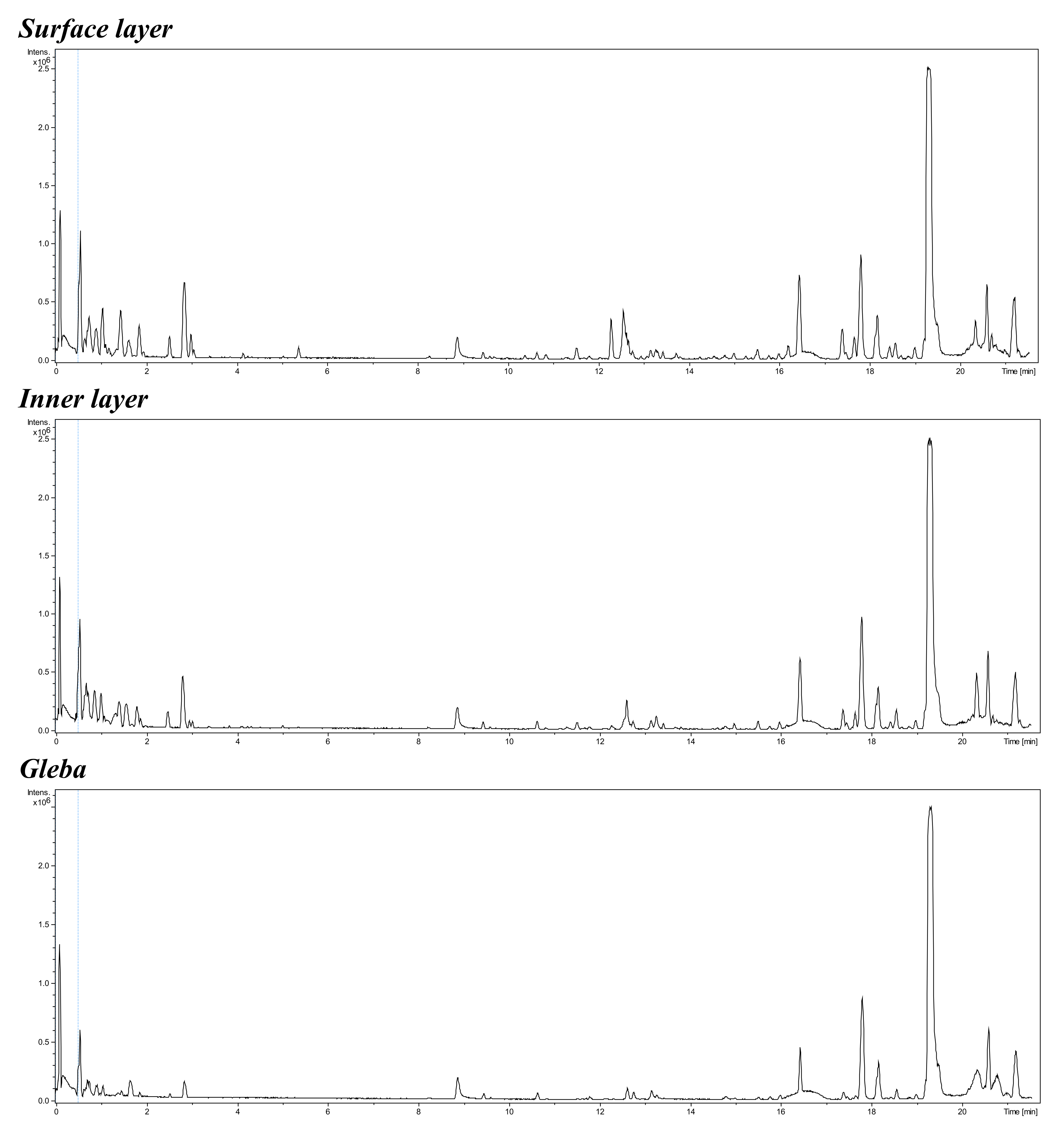

Supplement: Supplemental Information 5 — For extraction, 0.5 g of each truffle tissue sample was ground into a powder using a mortar and pestle with the addition of methanol (“Vecton”, Russia) in a 1:10 ratio (w/v; weight of tissue to volume of solvent). The resulting mixture was incubated for one hour on a roller mixer (MX-T6-S, BIOBASE, Jinan, China) and then centrifuged (LC-04A, Armed, Russia) at 3,000 rpm for 10 min. The supernatant was transferred into vials for chromatographic analysis. Chromatographic determination was performed using ultra-high-performance liquid chromatography (UHPLC) coupled with a triple quadrupole mass spectrometer (6470, Agilent Technologies, Germany) and an analytical Zorbax SB-Aq column (4.6 × 50 mm, 5 µm). The chromatographic conditions employed deionized water/formic acid (99.9:0.1, v/v) as solvent A and methanol/formic acid (99.9:0.1, v/v) as solvent B. Chromatographic separation of the target compounds was achieved using the following gradient program (time (min), %B): (0, 30%); (3, 10%); (4, 30%). The injection volume and flow rate were 3 µL and 0.8 mL/min, respectively. Data acquisition and processing were performed using MassHunter software (version B.08.00, Agilent Technologies, Germany). [file peerj-13-20037-s005.png]
